# Reports on the Hospitals of the United Kingdom

**Published:** 1910-02-26

**Authors:** Henry Burdett


### REPORTS
ON THE
### Hospitals of the United Kingdom.

By SIR HENRY BURDETT, K.C.B., K.C.V.O.

### XXVIII.—THE ROYAL BERKSHIRE HOSPITAL, READING.

This hospital was established in 1839. Most of the buildings are old, and, structurally, at the best, it cannot be classed as an up-to-date modern hospital. It is inconvenient to work, from the circumstance that when additions were made and extra wards built they were planned on a different level, so that staircases are numerous, and the working of the hospital and the moving of patients are rendered as troublesome as possible. For 70 years the older portions of the buildings have fulfilled their purposes well; but, as the administration block was designed for a hospital of 40 beds, and the institution now contains 168 beds, the general offices are not adequate, and much avoidable trouble must, we imagine, be caused to those responsible for the affairs of the hospital.

### Prepare for Rebuilding.

We may further say here, as the managers probably recognise, that the policy for them to pursue is to begin at once to accumulate funds to enable them to rebuild the hospital, and to provide that none of the immediate requirements shall be met by new buildings unless they are so placed as to enable them to be maintained when the day for rebuilding arrives. We, indeed, in view of the failures in London and elsewhere resulting from attempts to reconstruct when entire rebuilding would have been the cheapest and best course, would strongly advise the Committee to employ an architect under competent expert advice to lay out the whole of the land available, and to show by a block plan how the whole of it may be utilised to the best advantage when the time for rebuilding arrives. Such a scheme would show which portions can be best allocated at once as sites for a new casualty block and a new up-to-date home for nurses. The new nurses' home must be large enough to supply the needs of the existing and private nursing staffs, so as to secure that it may be capable of development to an extent equal to meet the demands of the county for private nurses. This latter development, in view of the present trend of legislation, should tend greatly to increase the income of the hospital from voluntary sources by giving each subscriber the privilege of being able to obtain at the hospital a private nurse, whose character can be guaranteed, whenever the needs of his family demand it.

### Thoroughness and Efficiency.

We visited this hospital in the evening, being late in arriving owing to bad weather, which delayed us *en route* for some hours. The matron, Miss Knowles, was unfortunately out, but we were taken round by Miss Wallace, who showed us everything most thoroughly. Indeed, her readiness to expose every corner, cupboard and drawer, and to show everything in detail with courageous promptitude exhibited such absolute confidence in the thoroughness and efficiency of the management that it gives us great pleasure to record it was abundantly justified. Most of the wards, as we have said, are old or relatively old; but the fittings have been renewed, and, so far as the space, which is rather limited, has made it possible the whole condition of the hospital from top to bottom is good.

### The Condition of the Wards.

The wards are in two parts. They are long and relatively narrow, and are rather sparsely furnished. New lockers and more chairs throughout the hospital would be an improvement. The wards are kept beautifully clean. The floors, furniture, and baths were spotless, and testify to the thoroughness of those responsible for the management and work. The same may be said of the operation theatre and its various sections, everything being kept admirably aseptic. Unfortunately, the floor of the theatre is terrazzo, and, as usual, it has cracked throughout nearly the entire length of the theatre, so that it is a danger as it exists at present, as German experience, especially, has proved. We should counsel the immediate renewal or removal of this floor, and the substitution of teak in narrow boards dovetailed together so as to form one of the best surfaces known for theatre floors.

### Wanted, a Modern Casualty Department.

We were informed that on some days in the week in the out-patient block, where casualties are treated, there are sometimes as many as from 12 to 20 cases of adenoids operated upon in a day. At present the accommodation is out of date, wholly inadequate for the work, and exhibits little regard for the comfort of the patients. It would be a relatively simple and inexpensive matter to reconstruct the buildings and to provide an up-to-date casualty department, with a small theatre and ample recovery-rooms. This work should be put in hand at once.

### Need for a Nurses' Home.

We have borne strong testimony to the marked efficiency of the matron's department and of the nurses. Such splendid service as they render demands that they should be properly housed and cared for. Too little consideration is shown them at present, for they are scattered about in three buildings: one of them, a so-called nurses' home, is too small and out-of-date, whilst some of the nurses were to be put at the top of the ward blocks then being reconstructed. Does not Reading contain one man of business who appreciates what is due to a large and efficient staff, who will make it his business not to rest until the Royal Berkshire Hospital has a new, well-planned, modern nurses' home of ample proportions, such as we have indicated above. We were amazed to find the nurses so badly provided for, for the Reading staff has a high reputation outside the hospital, and deserves better treatment than they experience in this respect at the hands of the committee and governors.

### Care and Economy in the Management.

We are glad to note that the Uniform System of Accounts is in use at this hospital, and that there is evidently great care in the management, a fact which no doubt contributes to its undoubted efficiency. The public have every reason to be satisfied with the working and administration, and we shall hope to find that the present deficiency of £2,500 between the ordinary income and the ordinary expenditure has been wiped out. The report presented to the Governors in February 1909 announced that plans had been prepared by Messrs. C. Smith and Son for extensive additions to the hospital, at the cost of £20,000. We hope that this expenditure will in no way cripple the possibilities of rebuilding, when the time comes, and that the advice we have ventured to give here may be followed in regard to an exhaustive scheme and plan, or large sums of money may be wasted at Reading, as they have been in the Metropolis and elsewhere.

### Mr. Herman Burney.

We were glad to find Mr. Herman Burney, B.A., Cantab., who was trained at the Seamen's Hospital, Greenwich, where he had an opportunity of thoroughly mastering modern hospital management, has settled down as Secretary, and is doing good work. His experience and knowledge should prove very valuable to the committee in the management of this important hospital.

---

The **Blackburn and East Lancashire Infirmary** will be reported on next week.

